# A Novel Method for Calibration of Digital Soil Images Captured under Irregular Lighting Conditions

**DOI:** 10.3390/s23010296

**Published:** 2022-12-27

**Authors:** Sung-Ha Baek, Ka-Hyun Park, Jun-Seo Jeon, Tae-Young Kwak

**Affiliations:** 1 School of Civil and Environmental Engineering & Construction Engineering Research Institute, Hankyong National University, Anseong 17579, Republic of Korea; 2 Department of Geotechnical Engineering Research, Korea Institute of Civil Engineering and Building Technology, Goyang 10223, Republic of Korea

**Keywords:** soil color, CIELAB color model, RGB color model, illuminance, color temperature

## Abstract

Soil color is commonly used as an indicator to classify soil and identify its properties. However, color-based soil assessments are susceptible to variations in light conditions and the subjectivity of visual evaluations. This study proposes a novel method of calibrating digital images of soil, regardless of lighting conditions, to ensure accurate identification. Two different color space models, RGB and CIELAB, were assessed in terms of their potential utility in calibrating changes to soil color in digital images. The latter system was determined to be suitable, as a result of its ability to accurately reflect illuminance and color temperature. Linear regression equations relating soil color and light conditions were developed based on digital images of four different types of soil samples, each photographed under 15 different light conditions. The proposed method can be applied to calibrate variations in the soil color obtained by digital images, thus allowing for more standardized, objective, and accurate classification and evaluation of soil based on its color.

## 1. Introduction

Because the color of soil varies depending on the constituent minerals, organic matter content, water content, and ion concentration, it has been widely used as a basic indicator to classify soil and predict its properties [1,2]. For example, in the field of agriculture, the soil color is used as a representative soil classification index [3], and different types of crops and farming types are monitored based on the soil color. In addition, in civil engineering, because similarly colored soils in adjacent areas are highly likely to have similar characteristics, the colors of the soil samples collected during ground surveys are recorded on the corresponding drilling logs.

Soil color is determined through observation with the naked eye. The Munsell soil color chart was developed to objectively distinguish soil colors observed using human vision, and represents colors as a combination of hue, value, and chroma [4]. ASTM [5] requires that the soil color be determined (indicated in the order of color brightness/saturation) by finding the standard color chip most similar to the color of the soil sample in the Munsell soil color book, which is the most widely used soil color determination method to date [6,7,8,9]. However, determining soil color using the Munsell soil color book has the following shortcomings:

1. When finding the standard color chip most similar to the color of the soil sample with the naked eye, the results are highly likely to be affected by the subjective perception or visual sensitivity of the observer, and the process is time-intensive [10];
2. Because the color of the soil sample and the standard color chip vary depending on environmental factors, such as illumination conditions, it is difficult to determine the absolute color [10,11];
3. Because the standard color chip of the Munsell book is divided into discrete elements, numerical and statistical analysis are difficult [12].

Digital image processing has recently received attention as a method to overcome this limitation [13,14,15]. Digital image processing entails a series of processes to obtain desired information by analyzing a digital image. A digital image consists of RGB (red, green, and blue) values from 0 to 255 assigned to each pixel (in the case of a black-and-white image, the pixels are instead assigned gray values from 0 to 255). Because digital image processing is a computerized process, it is possible to quickly and objectively determine soil color without the intervention of an observer, and because the color is displayed as a continuous value, numerical and statistical analysis are possible.

Accordingly, many researchers are conducting research to obtain soil color using digital image processing and to analyze the correlation between soil color and soil property. Persson [16], Zanetti et al. [17], Santos et al. [18], Park [19], and Kim [20] reported the RGB color intensity of soil obtained from digital images taken in an indoor studio and analyzed the water content (or moisture content), and Zhu et al. [21] analyzed the correlation between the gray color intensity and water content of soil obtained from black-and-white images. These studies revealed that the RGB color intensity and gray color intensity tend to decrease as the water content of the soil increases, and presented empirical equations capable of predicting the water content from the soil color. In addition, Gomez-Robledo et al. [22] and Moonrungsee et al. [23] obtained the RGB color intensity of soil from digital images taken with a smartphone camera. From those results, the soil color was matched to that of the Munsell soil color book [22] and was used to predict the content of phosphorus in the soil [23]. However, although the above studies were able to rapidly identify soil color based on digital image processing and successfully predict soil properties through this process, they had the limitation of using images taken in an indoor studio under constant light conditions. According to the principle of color expression, if the incident light changes, the soil color changes, so it is difficult to apply these research results to images taken under different light conditions, such as images taken at actual sites where it is impossible to control the light conditions.

In this study, a new method was explored to calibrate the changes in apparent soil color that occur according to changes in light conditions. An indoor studio that could simulate the characteristics of natural light (illuminance and color temperature) was established, and digital images were taken for four soil samples while changing the light conditions 15 times each (for a total of 60 shots). Digital image processing was performed to extract the soil colors of the photographed samples in two different color space models (RGB, CIELAB). We identified a color space model suitable for calibrating changes in soil color according to light conditions, and finally developed a new method for calibrating soil color in photographs taken under irregular light conditions.

The remainder of this paper is structured as follows. Section 2 describes our methodology, including the color space model, test equipment, digital imaging conditions, and soil specimens. Section 3 describes the analysis results, followed by a description of the proposed method. A conclusion is provided in Section 4.

## 2. Materials and Methods

### 2.1. Color Space Model for Digital Soil Images

Color is a three-dimensional psychophysical phenomenon that appears as light is incident on an object and reflected. In general, “light” refers to visible light, which is an electromagnetic wave with a wavelength between approximately 380 nm and 780 nm that can be seen with the naked eye. When light is incident on an object (incident light), light of some wavelengths is absorbed and light of other wavelengths is reflected (reflected light). The color is determined according to the wavelength of the reflected light. For example, plant leaves appear green because chlorophyll reflects electromagnetic waves in the green wavelength band (approximately 500 to 570 nm), and the soil color appears red because the soil particles reflect light in the red wavelength band (approximately 620 to 780 nm).

A method of numerically displaying the color of an object is called a color space model. A color space model represents a specific color as a point on a one-dimensional axis or in a three-dimensional space, and the method of defining a one-dimensional axis or three-dimensional space depends on the color space model (e.g., RGB, CIE XYZ, and CIELAB). Detailed definitions and theories of various color space models can be found in Billmeyer and Saltzman [24], Wyszecki and Stiles [25], Rossel et al. [26]. This chapter describes the two color space models (RGB and CIELAB) used in this study for soil color analysis.

**Figure 1:**
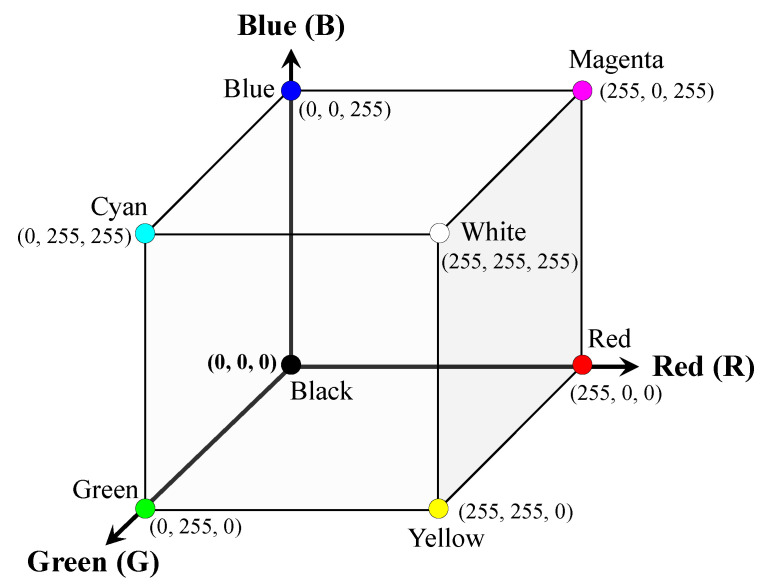
Three-dimensional RGB color space.

The RGB color system is the most widely used approach in electronic equipment, such as digital cameras, and expresses colors using the three primary colors of light: red (*R*), green (*G*), and blue (*B*). In other words, each color is expressed as a combination of red, green, and blue. When more red, green, and blue are applied, the resulting color is brighter; this is also called additive mixing. In the 8-bit digital system used by most electronic equipment, the RGB color intensity ranges from 0 (dark) to 255 (bright), and a total of 16,777,216 (=256^3^) colors can be expressed [25]. Figure 1 shows the RGB color space model. In a three-dimensional RGB color space, all colors correspond to a point within a cube whose vertices are red (255, 0, 0), green (0, 255, 0), blue (0, 0, 255), black (0, 0, 0), white (255, 255, 255), cyan (0, 255, 255), magenta (255, 0, 255), yellow (255, 255, 0).

The RGB color space model has the advantage of being able to reproduce most colors in a simple way. In addition, as mentioned above, because general electronic equipment (e.g., digital cameras, smartphones) used to acquire digital images adopts the RGB color space model, most existing studies [16,17,18,19,20,22,23] have obtained soil colors based on the RGB color space model.

However, the RGB color space model is not capable of representing all human-recognizable colors. In addition, there is a problem in that the wavelength indicated by these RGB values are different from the wavelengths recognized by the cone cells (short (S), medium (M), and long (L) wavelengths) responsible for human vision. Accordingly, in 1931, the Commission Internationale de l’Eclairage (CIE) established the CIEXYZ color space model, which can express all colors recognizable by humans based on the tristimulus values recognized by human cone cells [27]. However, it is difficult to use the CIEXYZ color space model in engineering calculations (numerical and statistical analysis of colors) owing to perceptual non-linearity in the method of expressing the distance between colors recognized by humans [25,26].

**Figure 2:**
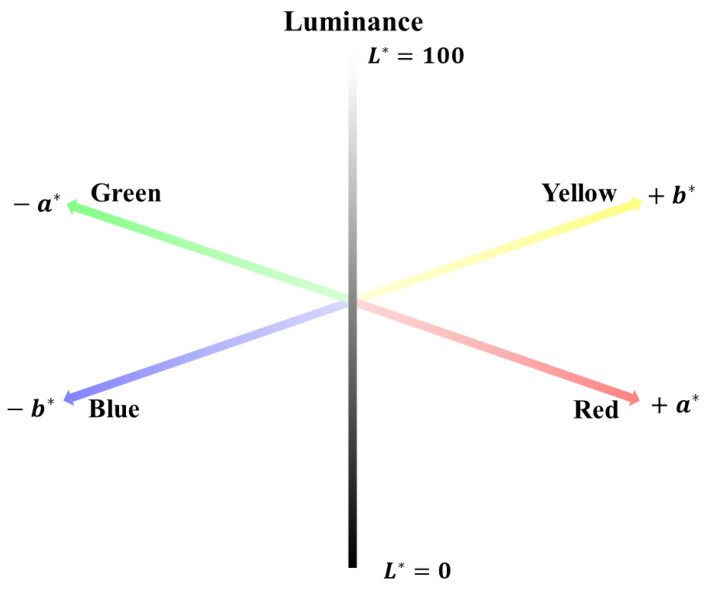
Three-dimensional CIELAB color space.

To overcome the above problem, CIE [28] proposed the CIELAB color space model, based on the CIEXYZ color space model. Unlike the CIEXYZ color space model, the CIELAB color space model is an almost perceptually uniform color space model [26]. In the CIELAB color space model, colors are expressed as combinations of L*, a*, and b*. L* represents the brightness of the color, expressed from 0 (dark) to 100 (bright). a* and b* are values representing the color, where a* indicates colors closer to red (positive) or green (negative), and b* indicates colors closer to yellow (positive) or blue (negative). Figure 2 shows the three-dimensional CIELAB color space, in which every color is represented by a point. Although the CIELAB color space model has not been widely applied to digital image analysis compared to the RGB color space model, the CIELAB color space model can express colors by dividing their components into “lightness” (L*-axis) and “chromaticity” (a*-b* plane). Therefore, it is expected to be useful in correcting changes in soil color resulting from light conditions (illuminance indicating the lightness of light and color temperature indicating the chromaticity of light).

In this study, to convert the digital image acquired based on the RGB color space model to the CIELAB color space model, a two-step conversion process was performed. First, the RGB color space model was converted to the CIEXYZ color space model using the color matching function of CIE [27], and the CIEXYZ color space model was converted to the CIELAB color space model through the conversion formula of CIE [28]. The color space model conversion process is described in more detail in a study by Rossel et al. [26].

### 2.2. Test Apparatus

**Figure 3:**
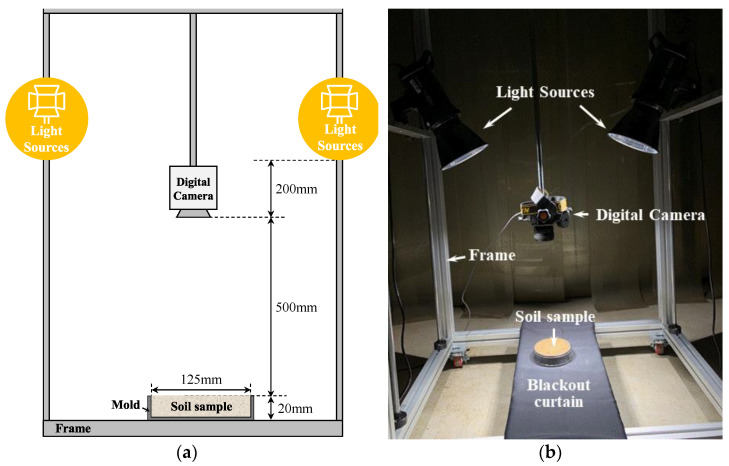
(**a**) Schematic diagram and (**b**) photograph of the digital image studio.

In this study, digital images were captured after creating a darkroom environment by completely blocking external light sources other than the lighting used in the experiment. As shown in Figure 3, the digital imaging equipment consisted of a digital camera, lighting, and a mold for soil samples. The digital camera, lighting, and mold were each fixed to a frame with an adjustable position. The distance between the imaging surface (soil sample surface) and the camera’s image sensor was kept constant at 500 mm with the focal length fixed (the camera’s autofocus function was not used), and the lighting was 700 mm from the imaging surface.

The digital camera used was a Nikon D850 with an AF-S 50 mm prime lens attached. This device can acquire 45.75-million-pixel digital images using a full-frame CMOS sensor, and minimizes camera shake due to influences, such as shutter operation. Two GODOX LED lights, SL100Bi, were used for lighting; based on the imaging surface, the color temperature could be adjusted from 2800 K to 6000 K and the illuminance could be adjusted up to 65,000 lux. The mold containing the soil sample was made of matte black plastic with an inner diameter and height of 125 mm and 20 mm, respectively. A blackout curtain was placed on the bottom surface where the mold was placed to minimize the effect of light reflected from objects other than the sample. In addition, image distortion was minimized by arranging the image-taking surface in the vertical direction with the digital camera.

### 2.3. Soil Specimen

In this study, four soil samples (Jumunjin sand, Anseong soil, Yongin soil, and Gwanak soil) with different colors were used, according to the location of collection and differences in the constituent minerals. Jumunjin sand is a silica-based sand collected from Jumunjin Beach in Gangneung, Republic of Korea, and has a bright yellow color. Anseong soil and Yongin soil are weathered granite soil collected from borrow pit located in Anseong and Yongin, respectively, and Gwanak soil is weathered granite soil collected from Mt. Gwanak in Seoul, Republic of Korea.

**Figure 4:**
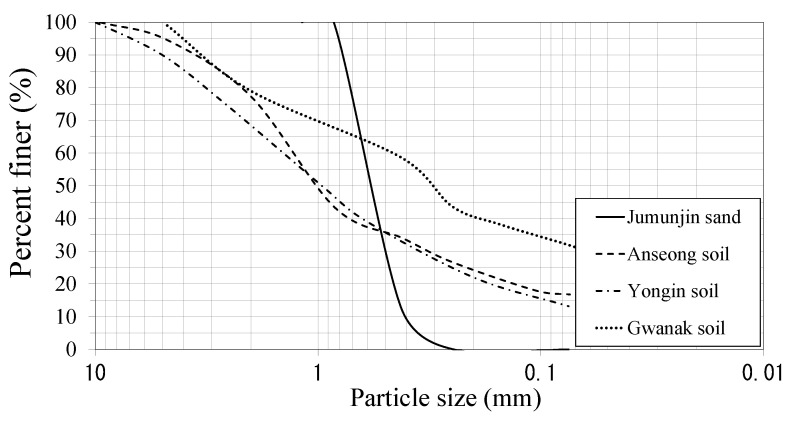
Particle size distribution of the test specimens.

In accordance with ASTM [29], mechanical sieve analysis was performed to analyze the particle size of four soil samples (see Figure 4). Jumunjin sand was classified as poorly graded sand (SP) by the Unified Soil Classification System (USCS) and had an average effective particle diameter (*D*_50_) of 0.58 mm. Anseong soil, Yongin soil, and Gwanak soil were classified as SM (silty sand) according to the USCS. The average effective particle diameters (*D*_50_) of the three soil samples (Anseong soil, Yongin soil, and Gwanak soil) were 1.05 mm, 0.96 mm, and 0.32 mm, respectively, and the passing percentages of 200 sieves (the ratios of particles with a diameter of 0.074 mm or less) were 16.8%, 13.2%, and 31.9%, respectively.

Four soil samples dried at 110 ± 5 °C for more than 24 h were compacted into cylindrical molds to prepare the samples for photography. The samples for photography each had a dry density corresponding to 70% of the relative density of each sample (the dry densities of Jumunjin sand, Anseong soil, Yongin soil, and Gwanak soil are 1.52 g/cm^3^, 1.73 g/cm^3^, 1.71 g/cm^3^, and 1.73 g/cm^3^, respectively). A compactor rod with the same diameter as the inner diameter of the mold was used so that each sample was homogeneously and flatly compacted.

### 2.4. Test Conditions

In this study, digital images were captured after simulating natural light conditions using lighting in a dark room completely blocked from external light sources. In this case, “natural light conditions” refers to illuminance and color temperature, which are indicators that quantitatively represent the lightness and chromaticity, respectively, of the light source. Table 1 shows the illuminance and color temperature of natural light measured in Goyang-si, Gyeonggi-do, Republic of Korea from 15 February 2022 to 28 March 2022 using Konica Minolta’s portable optical property measuring device CL-200A. The illuminance of natural light was in the range of 15,540–65,040 lux, and showed a tendency to decrease from morning to afternoon and during cloudy weather. In addition, the color temperature of natural light was in the range of 3590–5808 K and showed smaller values close to sunset. The measured color temperature showed a range similar to that measured by Jeon et al. [30] for four seasons from April 2017 to April 2018.

Based on the measurement results of the illuminance and color temperature of natural light, the light conditions to be applied when taking digital images of soil samples were determined as shown in Table 2. For the four soil samples (Jumunjin sand, Anseong soil, Yongin soil, and Gwanak soil), digital images were captured while changing the light conditions 15 times (60 shots in total). Light with a target illuminance (15,000 lux, 35,000 lux, 50,000 lux, and 65,000 lux) and color temperature (3000 K, 3800 K, 4500 K, and 5500 K) was irradiated onto the sample using the LED lights. At this time, slight errors occurred in the process of manually adjusting the illuminance and color temperature of the lighting (average errors of illuminance and color temperature were 1.57% and 1.05%, respectively). Therefore, before taking an image of the soil sample, we measured the illuminance and color temperature of the soil sample actually received from the image-taking surface through using the optical property measuring device, and the measured values were used to analyze the results.

Because digital camera settings (aperture value, shutter speed, ISO, white balance, flash, etc.) have a great influence on digital image measurement results, it is necessary to set appropriate values according to the shooting conditions and subjects. In this study, the aperture, shutter speed, and ISO values were set to f/5, 1/1000 s, and 200, respectively, considering the size and shooting distance of the soil sample. The white balance was fixed at 5500 K and the picture was taken with the flash off, so the influence of the camera settings on the image measurement results was excluded.

## 3. Results and Discussion

### 3.1. Digital Soil Images

The soil color change according to light conditions was analyzed based on the digital image capture results of soil samples. Because the trend of soil color change according to light conditions was similar regardless of the soil samples, digital images of Yongin soil and Gwanak soil are shown as representative examples.

**Figure 5:**
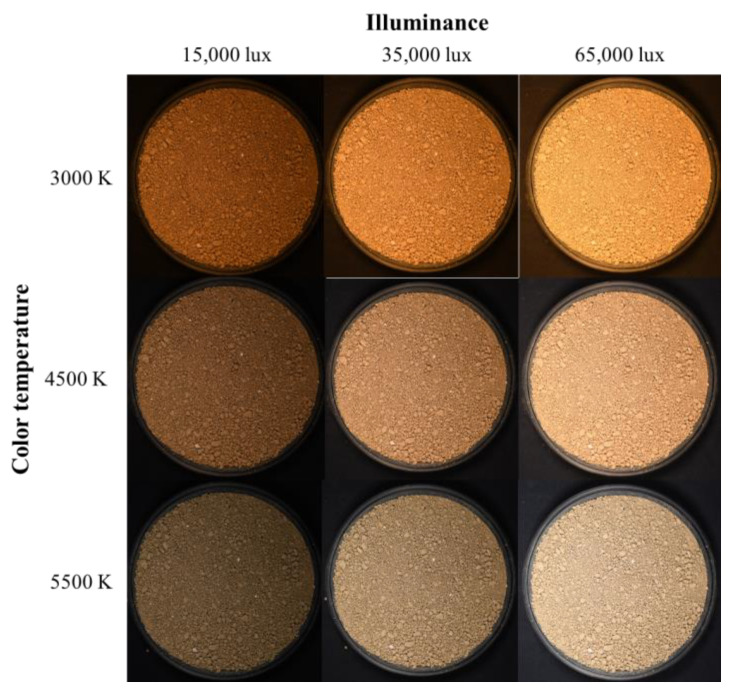
Digital images of Yongin soil.

**Figure 6:**
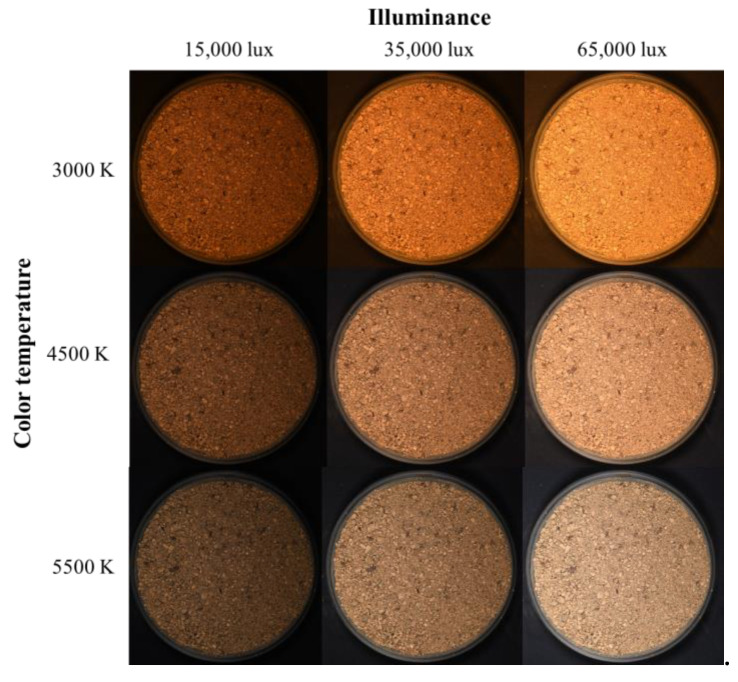
Digital images of Gwanak soil.

Figure 5 and Figure 6 show digital images of Yongin soil and Gwanak soil, respectively, taken under three different illuminance (15,000 lux, 35,000 lux, and 65,000 lux) and color temperature (3000 K, 4500 K, and 5500 K) conditions. Even though the identical soil samples were photographed using the same camera settings, the soil color displayed on the image varied greatly depending on the light conditions. This is because, as described above, when the incident light is changed according to the color expression principle, the reflected light that determines the color also changed.

The color temperature is a numerical representation of the chromaticity of the light source using absolute temperature (K). The color temperature of a red light source is lower, and the color temperature of a blue light source is higher. The illuminance of the light source is a value indicating the intensity of light received by a specific surface; the higher the illuminance, the brighter the light source. Each soil color also showed the same trend regarding the changes in the light source according to color temperature and illuminance (see Figure 5 and Figure 6). That is, as the color temperature of the lighting increased, the soil’s color became closer to blue than red, and as the illuminance increased, it became brighter. The phenomenon of soil color change depending on lighting illustrates the limitations of existing studies [16,17,18,19,20,21,22,23].

The above studies suggested a method of predicting soil property using soil color obtained based on digital image processing, but were conducted based on images taken in an indoor studio under constant light conditions. The suggested research results cannot be applied in outdoor environments where the light conditions do not match those used in the studies, or where it is practically impossible to control the light conditions. In order to make the above research results more versatile and applicable in the field, a digital image processing-based soil color analysis method that can consider irregular light conditions (color temperature and illuminance) is required.

The next section describes the digital image processing performed to numerically acquire the soil colors of the samples in two color space models (RGB and CIELAB). The color change according to the light conditions was analyzed, and a color space model capable of considering irregular light conditions was confirmed.

### 3.2. Soil Color Based on Digital Image Processing

**Figure 7:**
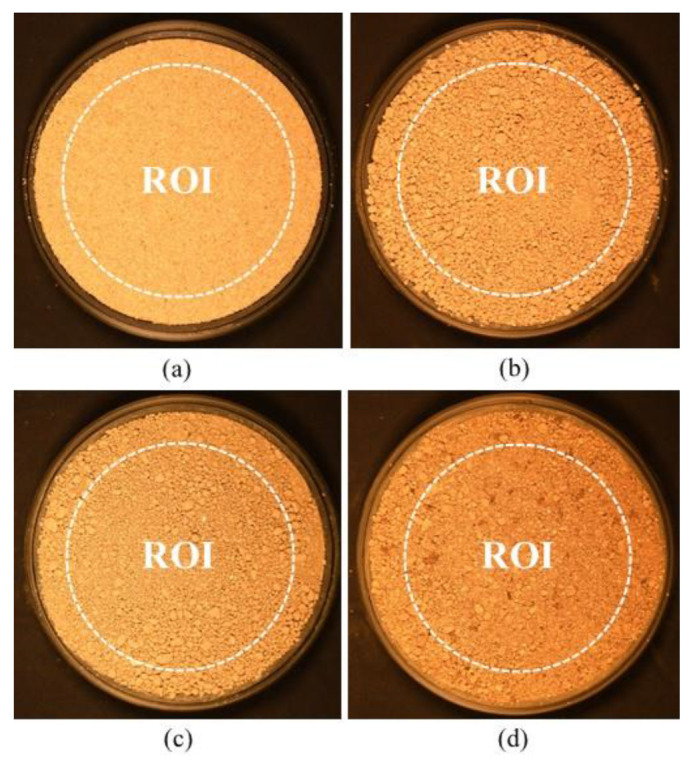
Region of interest (ROI) in digital image analysis: (**a**) Jumunjin sand, (**b**) Anseong soil, (**c**) Yongin soil, and (**d**) Gwanak soil.

In this study, digital image processing was performed in three major steps (region of interest (ROI) setting, color space model conversion, and color extraction within the region of interest). As shown in Figure 7, the ROI was set as a circle with a diameter of 100 mm that shared its center with the soil sample (the diameter of the soil sample was 125 mm). Colorimetric conversion was performed according to the procedure described in Section 2. Referring to Gomez-Robledo et al. [22], the statistical mode (hereinafter referred to as “mode”) was used as a representative value of soil color in the ROI. This is because Garcia et al. [31] demonstrated that the mode is more suitable than the mean value for the analysis of heterogeneous color images (here, soil images), thereby eliminating the undesirable effect of noise.

Simple linear regression analysis was performed using light conditions (illuminance and color temperature) as independent variables (explanatory variables) and soil color intensity as dependent variables. Linear regression analysis is usually performed unless there is clear evidence that a non-linear relationship (e.g., polynomial relationship, exponential regression, logarithmic regression) exists between the independent and dependent variables. There is no clear evidence to indicate a non-linear relationship between the light condition and the soil color intensity; also, there is not enough data accumulated to perform a non-linear regression analysis. As a performance indicator of the simple linear regression model, the coefficient of determination (*R*^2^) that the ratio of the variance of the dependent variable explained by the independent variable in the linear regression analysis using the least squares method was used.

**Figure 8:**
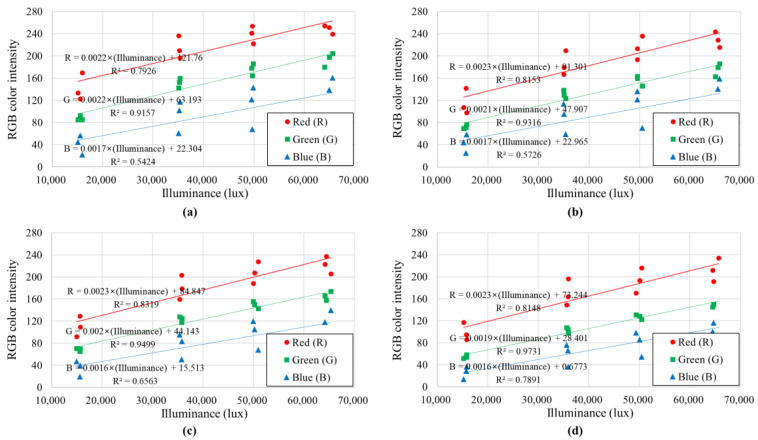
Mode RGB color intensities according to the illuminance of light: (**a**) Jumunjin sand, (**b**) Anseong soil, (**c**) Yongin soil, and (**d**) Gwanak soil.

**Figure 9:**
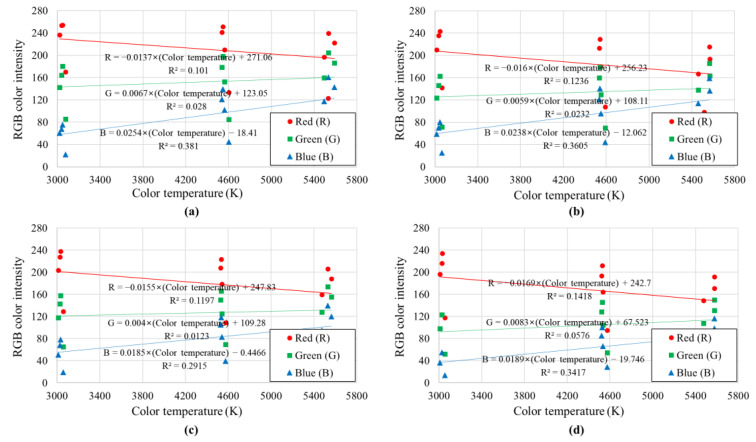
Mode RGB color intensities according to the color temperature of light: (**a**) Jumunjin sand, (**b**) Anseong soil, (**c**) Yongin soil, and (**d**) Gwanak soil.

Figure 8 and Figure 9 show the mode values of soil colors (*R*, *G*, and *B*) derived for the RGB color display system with respect to the illuminance and color temperature, respectively, of the light received by the soil. As shown in Figure 8, in all four soil samples, as the illuminance increased, the RGB color intensity of the soil tended to increase. In the case of green (*G*), which is known to have the highest correlation with brightness among the three colors of RGB, the correlation coefficient with illuminance (*R*^2^) was higher than 0.91, but the correlation coefficients with red (*R*) and blue (*B*) were relatively low. This is because the correlation coefficient is relatively low compared to the illuminance, but the color temperature also affects the soil’s colors (especially the intensity of red (*R*) and blue (*B*)). As can be seen in Figure 9, the red intensity of the soil sample photographed under lighting with a low color temperature (red series) was high, and the blue intensity of the soil sample photographed under the lighting with a high color temperature (blue series) was high.

One interesting finding regarding the relationship between RGB color intensity and illuminance is that the linear regression equations of the four soil samples with different constituent minerals had similar slopes (see Figure 8). Because there were differences in the soil color of the four samples, there were differences in the *y*-intercept of the linear regression equation, but there was no significant difference in the degree of soil color change (i.e., slope) according to the change in illuminance. Nevertheless, the soil color displayed by the RGB color display system is affected by both illuminance and color temperature; when using the RGB color space model, it would be difficult to calibrate (or exclude) the effects of illuminance and color temperature.

**Figure 10:**
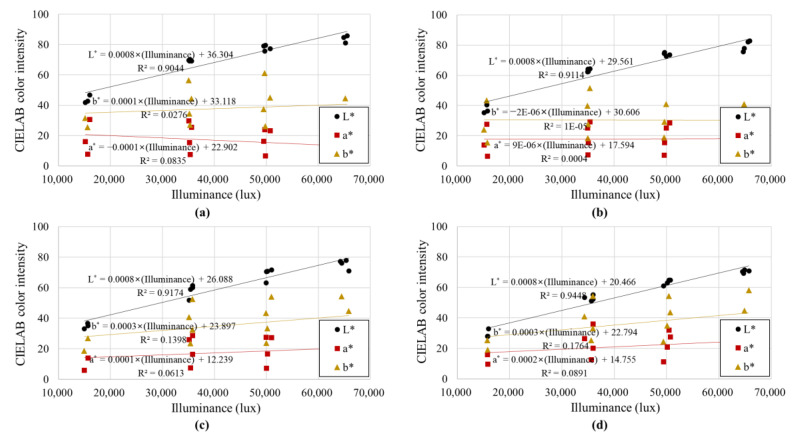
Mode CIELAB color intensities according to the illuminance of light: (**a**) Jumunjin sand, (**b**) Anseong soil, (**c**) Yongin soil, and (**d**) Gwanak soil.

**Figure 11:**
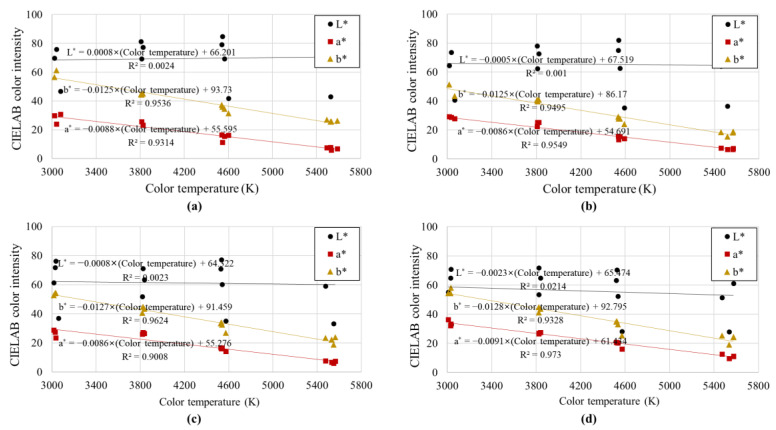
Mode CIELAB color intensities according to the color temperature of light: (**a**) Jumunjin sand, (**b**) Anseong soil, (**c**) Yongin soil, and (**d**) Gwanak soil.

Figure 10 and Figure 11 show the mode values of soil colors (L*, a*, and b*) derived for the CIELAB color display system for the illuminance and color temperature, respectively, of the light received by the soil. In all four soil samples, L* showed a high correlation with illuminance, and a* and b* showed a high correlation with color temperature (*R*^2^ > 0.90). In contrast, illuminance had little effect on a* and b*, and color temperature had little effect on L*. This is because the CIELAB color space model has the characteristic of expressing colors by dividing them into “lightness” (L*-axis) and “chromaticity” (a*-b* plane). In the CIELAB color space model, L* is an index indicating the lightness of a color, so L*, the lightness of the soil color, increased as the illuminance increased. As the color temperature increased and changed from red to blue, the soil color moved away from red and closer to blue, and the color indicators a* (red to green) and b* (yellow to blue) gradually decreased (see Figure 2). Furthermore, similar to the relationship between RGB color intensity and illuminance, the linear regression slopes of (1) L* and illuminance, and (2) a*, b* and color temperature were similar regardless of the type of soil sample. Because the soil colors of the four samples were different, there were differences in the *y*-intercept of the linear regression equation, but there was no significant difference in the degree of change in soil color according to changes in illuminance and color temperature.

In summary, among the color components, L* was affected only by illuminance, but a* and b* were affected only by color temperature, and the correlation was high. Therefore, based on the relationships between (1) illuminance and color temperature and (2) illuminance and color temperature obtained based on the CIELAB color space model, the effects of illuminance and color temperature on soil color can be calibrated (or excluded). It is thought that this goal can be achieved using our novel calibration method, which is described in more detail in the following subsection.

### 3.3. Digital Soil Image Calibration Method

Through digital image analysis of soil samples captured under various light conditions, there is a linear relationship between the soil color displayed based on the CIELAB color space model and the illuminance and color temperature of the light received by the soil, as follows (see Figure 10 and Figure 11):

(1) $$L^{*}=a_{L}I+f_{L},$$

(2) $$a^{*}=a_{a}T+f_{a},$$

(3) $$b^{*}=a_{b}T+f_{b}.$$

Here, *I* and *T* denote the illuminance and color temperature, respectively, of the light received by the soil. *a_L_* and *f_L_* are the slope and intercept of the linear regression equation between the L* value of the soil color and illuminance, respectively; *a_a_* and *f_a_* are the slope and intercept of the linear regression equation between the a* value of the soil color and color temperature, respectively; and *a_b_* and *f_b_* are the slope and intercept of the linear regression between the b* value of the soil color and the color temperature. The digital soil image calibration procedure through the above formula is as follows:

1. The target soil is sequentially irradiated by various configurations of light with four or more different illuminances and color temperatures, and digital images are taken. At this time, the light intensity and color temperature (i.e., *I* and *T*) of the target soil are recorded;
2. From the digital soil images, the soil color (i.e., L*, a*, and b*) of the target soil is obtained based on the CIELAB color space model;
3. A linear regression analysis of the relationship between the light conditions and soil color is plotted on a two-dimensional plane (*I*-L* plane, *T*-a* plane, and *T*-b* plane); using Equations (1)–(3), the slope (*a_L_*, *a_a_*, and *a_b_*) and intercept (*f_L_*, *f_a_*, and *f_b_*) are found.

Through the relational expression between the light condition and soil color determined in this way, the soil color of the target soil can be obtained under a given light condition (substituting the given light condition into Equations (1)–(3)).

Table 3 shows the slope of the linear regression equation between L* and illuminance, as well as that between a* and b* and color temperature, which showed a particularly significant correlation coefficient among the relationships between light conditions and soil color (see Figure 10 and Figure 11). As mentioned earlier, the slopes of the linear regression equations in Figure 10 and Figure 11 were similar regardless of the type of soil sample. When the average value of the linear regression slope is substituted into the slopes (i.e., *a_L_*, *a_a_*, and *a_b_*) of Equations (1)–(3), the linear relationship between soil color and light conditions is as follows:

(4) $$L^{*}=0.0008I+f_{L},$$

(5) $$a^{*}=-0.0126T+f_{a},$$

(6) $$b^{*}=-0.0088T+f_{b}.$$

Equations (4)–(6) are relational expressions between light conditions and soil color that can be applied to soils similar to the samples used in this study (silica-based sand or weathered granite soil). Through these equations, the relationship between light conditions and soil color can be determined from just one digital image capture. The procedure is as follows:

1. Take a digital image of the target soil, and record the light intensity and color temperature (i.e., *I* and *T*) of the target soil;
2. Obtain the soil color (i.e., L*, a*, and b*) of the target soil based on the CIELAB color space model;
3. Substitute in Equations (4)–(6) to find the intercepts (*f_L_*, *f_a_*, and *f_b_*).

By substituting the given illuminance and color temperature into the relationship between light conditions and soil color determined through this process, the soil color under specific light conditions can be calculated.

As mentioned earlier, previous studies [16,17,18,19,20,21,22,23] proposed a correlation between soil color and soil property based on digital images taken in an indoor studio under constant light conditions. It should be noted that if the light conditions are different, the soil color will be different, even for the same soil. Thus, to utilize the previously proposed correlation between soil color and soil property, it is necessary to obtain the soil color by creating the same light conditions as in previous studies and then capturing digital images. However, it is difficult to create light conditions identical to those in the previous studies in an indoor studio, and in particular, it is virtually impossible to control the light conditions in an outdoor case.

From this point of view, the proposed soil color calibration method has great significance. If Equations (1)–(3) or Equations (4)–(6) are determined based on the digital image of a soil sample taken under arbitrary light conditions, the soil color under a specific light condition can be calculated. First, to establish the correlation between soil color and soil property, the soil colors (i.e., L*, a*, and b*) obtained based on the CIELAB color space model are used. At this time, the illuminance and color temperature of the light received by the soil sample are recorded. Then, to predict the soil properties, Equations (1)–(3) or Equations (4)–(6) are determined using the digital images of soil samples taken under arbitrary light conditions. The soil color is obtained by substituting the specific light conditions (illuminance and color temperature of light received by soil samples when establishing the correlation between soil color and soil property) into the aforementioned formulas; this is used to predict the soil property.

According to the above process, it is possible to calibrate the soil color photographed under irregular light conditions to the corresponding color under the desired light condition. It is expected that this approach will allow soil color to be used as a reliable indicator for predicting soil properties, overcoming the limitations of existing studies resulting from variation in soil color due to light conditions.

## 4. Conclusions

In this study, a new method was proposed to calibrate the color of soil, which changes according to different light conditions. Digital images were captured while changing the light conditions 15 times for 4 soil samples. The soil color of the samples photographed through digital image processing was extracted according to two color space models (RGB and CIELAB), and the color change according to the light conditions was analyzed. Through this process, the following conclusions were reached.

1. Even when the uniformly formed soil samples were photographed with the same camera settings, the soil color varied greatly depending on the color temperature and illuminance of the light received by the soil. Most existing digital image processing-based soil color analysis studies were performed under constant light conditions, making them less applicable to outdoor environments with different light conditions, or environments in which control of light conditions is virtually impossible;
2. Color analysis based on the RGB color space model indicated that the color parameters (*R*, *G*, and *B*) increased as the illuminance increased. Among these parameters, green (*G*), which is known to have the highest correlation with brightness, had a very high correlation with illuminance, but red (*R*) and blue (*B*) showed relatively low correlations due to the effect of color temperature. It was concluded that it would be difficult to exclude (or calibrate) the influence of light conditions through this approach, as the soil color displayed by the RGB color display system is partially affected by both illuminance and color temperature;
3. The soil color analysis based on the CIELAB color space model showed that L* was affected only by illuminance, but a* and b* were affected only by color temperature, and the correlation was high. This is because L* represents the lightness of a color and a* and b* are indices representing the chromaticity. Using the relationship between illuminance and color temperature, as well as that between illuminance and color temperature, obtained based on the CIELAB color indicator, it is possible to calibrate (or exclude) the effect of illuminance and color temperature on soil color;
4. Using the linear regression equation of soil color and light conditions based on the CIELAB color space model, a method for calibrating soil color was proposed. Through the proposed method, the soil color of soil samples photographed under arbitrary light conditions can be corrected to the soil color under the desired light conditions.

By excluding the effect of light conditions on soil color using the method proposed in this study, soil color can be used as a reliable indicator for predicting soil properties, such as constituent minerals, organic matter content, water content, and ion concentration. The proposed method makes the soil color obtained from the digital images more versatile and applicable in the field; thus, digital image processing will be a simple, fast, and inexpensive method of on-site analysis for land and crops. However, the above results are derived based on digital images taken for four soil samples (silica-based beach sand and weathered granite soil). To make the conclusion more convincing, the proposed soil color calibration process needs to be supplemented by acquiring soil color from different soil samples (with different or similar mineral composition). It is necessary to analyze the correlation between soil color and light conditions according to the framework established in this study using more diverse soil samples.

## Figures and Tables

**Table 1:** Illuminance and color temperature of natural light in Goyang-si, Gyeonggi-do, Republic of Korea (15 February 2022–28 March 2022).

Date	Time	Weather	Direct Light *	Illuminance (lux)	Color Temperature (K)
02/15/2022	13:30	Clear	O	65,040	5350
02/16/2022	17:00	Clear	O	33,730	3762
02/17/2022	14:30	Clear	O	47,580	5237
02/18/2022	10:30	Clear	O	58,530	5353
02/20/2022	10:30	Clear	O	60,200	5551
02/21/2022	14:30	Cloudy	X	15,540	5808
02/22/2022	10:30	Clear	O	60,100	5255
03/03/2022	10:30	Clear	O	59,520	5312
03/08/2022	14:30	Cloudy	X	16,700	5252
03/08/2022	14:30	Cloudy	O	43,720	5122
03/11/2022	17:00	Cloudy	O	28,720	3640
03/15/2022	14:30	Cloudy	O	45,570	5605
03/22/2022	10:30	Clear	O	49,200	5342
03/22/2022	17:00	Clear	O	32,750	3811
03/27/2022	14:30	Clear	X	16,210	5355
03/28/2022	17:00	Clear	O	35,420	3590

* “O” represents the direct sunlight condition, whereas “X” represents the shaded condition.

**Table 2:** Lighting conditions for digital soil image acquisition.

Illuminance (lux)	Color Temperature (K)
15,000	3000
	4500
	5500
35,000	3000
	3800
	4500
	5500
45,000	3000
	3800
	4500
	5500
65,000	3000
	3800
	4500
	5500

**Table 3:** Slope of linear regression lines between lighting conditions (illuminance and color temperature) and CIELAB-based soil color.

Soil Type	*a_L_*	*a_a_*	*a_b_*
Jumunjin sand	0.0008	−0.0125	−0.0088
Anseong soil	0.0008	−0.0125	−0.0086
Yongin soil	0.0008	−0.0127	−0.0086
Gwanak soil	0.0008	−0.0128	−0.0091

## Data Availability

The data presented in this study are available on request from the corresponding author.
